# An Operational Tool for the Automatic Detection and Removal of Border Noise in Sentinel-1 GRD Products

**DOI:** 10.3390/s18103454

**Published:** 2018-10-14

**Authors:** Mattia Stasolla, Xavier Neyt

**Affiliations:** Signal & Image Center, Royal Military Academy, B–1000 Brussels, Belgium; xavier.neyt@rma.ac.be

**Keywords:** Sentinel-1, Synthetic Aperture Radar (SAR), Ground Range Detected (GRD), border noise, mathematical morphology

## Abstract

The presence of border noise in Sentinel-1 Ground Range Detected (GRD) products is an undesired processing artifact that limits their full exploitation in a number of applications. All of the Sentinel-1 GRD products generated before March 2018—more than 800,000—are affected by this particular type of noise. In March 2018, an official fix was deployed that solved the problem for a large portion of the newly generated products, but it did not cover the entire range of products, hence the need for an operational tool that is able to effectively and consistently remove border noise in an automated way. Currently, a few solutions have been proposed that try to address the problem, but all of them have limitations. The scope of this paper is therefore to present a new method based on mathematical morphology for the automatic detection and masking of border noise in Sentinel-1 GRD products that is able to overcome the existing limitations. To evaluate the performance of the method, a detailed numerical assessment was carried out, using, as a benchmark, the ‘Remove GRD Border Noise’ module integrated in ESA’s Sentinel Application Platform. The results showed that the proposed method is capable of very accurately removing the undesired noisy pixels from GRD images, regardless of their acquisition mode, polarization, or resolution and can cope with challenging features within the image scenes that typically affect other approaches.

## 1. Introduction

The Sentinel-1 space mission was launched on 3 April 2014 by the European Space Agency (ESA) under the framework of the Copernicus programme. It consists of a constellation of two polar-orbiting satellites that mount a C-band Synthetic Aperture Radar (SAR) imaging system, enabling all-weather and day-and-night mapping of the Earth at a high temporal resolution [1]. The Sentinel-1 products that are intended for the majority of the users, and therefore, generally made available, are Level-1 data, provided as Single Look Complex (SLC) and Ground Range Detected (GRD) products. In particular, GRD products are focused data that have been detected, multi-looked, and projected to ground range. The three most common acquisition modes for Sentinel-1 GRD products are Interferometric Wide swath (IW), Extra-Wide swath (EW), and Stripmap (SM); the two typically available spatial resolutions are high (H) and medium (M); the polarization can be either single (SSH, SSV) or dual (SDH, SDV) [2]. A summary of the characteristics of the main GRD products is provided in Table 1.

**Figure 1:**
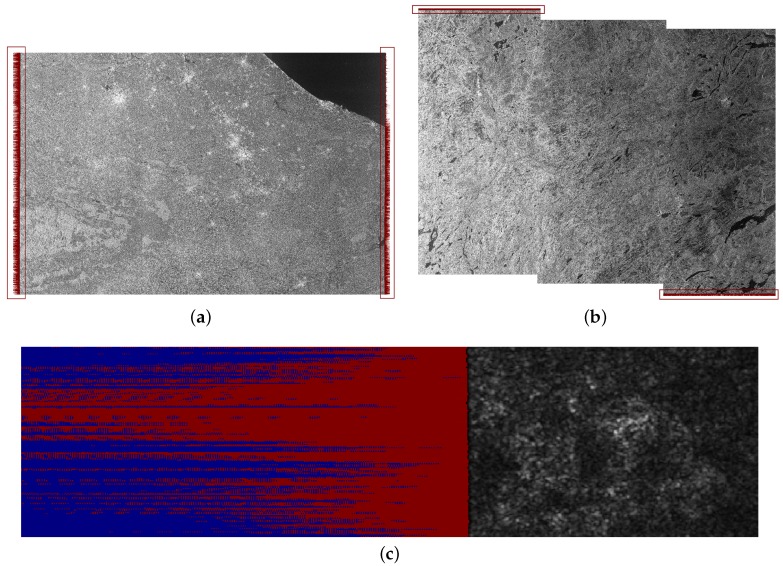
Spurious dark pixels generated at the borders of Sentinel-1 GRD images, highlighted in red: (**a**) cross-track border noise in the range direction (left and right); (**b**) along-track border noise in the azimuth direction (top and bottom); (**c**) close-up of the border noise, highlighted in red, mixed with samples correctly set to zero by the Instrument Processing Facility (IPF), highlighted in blue. Sentinel-1 images ©Copernicus 2018.

The transformation of raw data into Level-1 products is performed by the Sentinel-1 Instrument Processing Facility (IPF), a key component that has been constantly updated and improved [3]. All the Level-1 GRD products generated by the IPF from its very first version, 2.34, to version 2.84 are known to be affected by undesired dark (non-zero) samples at the image borders [4]. More specifically, the products are affected by cross-track border noise in the range direction and along-track border noise in the azimuth direction [5]. Two examples are provided in Figure 1a,b, where the border noise (left/right and top/bottom) has been highlighted in red. Figure 1c shows, in more detail, the pattern of the border noise (in red) that is often mixed with samples correctly set to zero by the IPF (in blue). As can be seen from the examples, this pattern is not constant and varies from image to image, making it difficult to systematically remove it.

This issue has clearly hindered the full exploitation of Sentinel-1 data and has impacted many applications, such as ship detection [6], wind speed retrieval [7], and sea ice detection [8]—where fluctuations in the background distribution caused by imhomogeneities (e.g., sea-clutter, beam seams) typically raise the number of false alarms—, or the automatic generation of stacks of images and mosaics—where data gaps should be avoided [9]. For these reasons, version 2.90 of the IPF was deployed on 13 March 2018 to address the problem [4] and since then, the newly generated IW and EW products have all of the no-value pixels at the image borders correctly set to zero. Although these are the majority of the available GRD products, the IPF upgrade did not completely solve the problem. First of all, the upgrade did not concern the SM products. If we consider that, on average, Sentinel-1 acquires seven SM images per day, the overall number of products involved becomes non-negligible. Secondly, more than 800,000 products generated from April 2014 to March 2018 have undesired noise at the image borders and need to be processed in order to remove it.

As of this moment, the only generally available tool that could potentially accomplish this task is the ‘Remove GRD Border Noise’ module that is integrated in ESA’s Sentinel Application Platform (SNAP) [10]. It implements a masking approach that exploits the denoising vectors on the co-polarisation channel (HH or VV) in order to set the undesired dark pixels to zero and then remove them by thresholding [4]. Nevertheless, the procedure has some limitations, as it requires the level of signal to always be higher than the instrument noise floor. This means that it is typically effective over land, but it does not properly work over water body areas, especially at low wind conditions [4]. When SNAP’s ‘Remove GRD Border Noise’ is not effective, a reported workaround is to crop image borders [11]. As a matter of fact, this cannot be seen as a systematic solution, as the pattern of border noise is not regular and can change from image to image. The amount of data loss due to the cropping based on a fixed window would be significant. Therefore, neither of the two options could be considered for the automated processing of Sentinel-1 data and alternatives must be found.

In the literature, only three papers have been published that have tried to address the problem. In [12], Dyatmika et al. presented a method that first thresholds the VH and VV bands of a IW GRD product by applying two fixed thresholds and then segments the noise to remove big areas. The method was tested on two images and assessed by visual interpretation. It only focused on near range and far range border noise, with no mention of the border noise in the azimuth direction. In [9], a border noise removal tool was implemented within the automated mosaicking system of the Canadian Ice Service (CIS). The method is based on a four-round scanning that identifies the data/noise border based on the adjacent pixels ratio. The method’s validation was provided in the form of visual evaluation of generated arctic ice mosaic employing 450 Sentinel-1 scenes acquired in November 2017. Finally, in [5], Ali et al. presented, in detail, the problem and proposed three different methods to address it for the case of IW GRDH products. The first one was an anomaly detection approach based on the interquantile range (IQR) Tukey test; the other two were bidirectional sampling methods (in slightly different formulations). All of them created a mask of the border noise based on the co-polarization channel. The assessment was carried out over a large number of images, but the results were generically provided in terms of the number of images with fully removed border noise.

According to the discussion above, the presence of border noise in GRD images is certainly a limiting factor for the full exploitation of Sentinel-1 data and needs to be solved by developing an automatic tool that is capable of consistently masking the no-value pixels. Although some attempts have been done to this end, there still remain important open issues:

(i) The new methods for noise border removal have only focused on IW products. This means that for EW, and especially for SM products that have not been concerned by the IPF upgrade to version 2.90, no solutions are currently available;
(ii) The existing results are limited to high resolution images, as this is the typical format of IW products. Further tests are therefore required for medium resolution images;
(iii) No detailed quantitative performance assessment has ever been carried out to understand the actual impact of border noise on Sentinel-1 image quality.

The scope of this paper is therefore to fill these gaps. First, we describe a novel approach based on mathematical morphology that allows us to automatically and consistently remove the border noise from Sentinel-1 GRD products. The algorithm can be applied to any image, regardless of its acquisition mode, spatial resolution, or polarization. Secondly, we provide a very detailed performance assessment that numerically shows how much the border noise can affect the quality of Sentinel-1 images and how accurately our method is able to mask it. A comparison with SNAP’s ‘Remove GRD Border Noise’ completes the analysis.

## 2. Methodology

**Figure 2:**
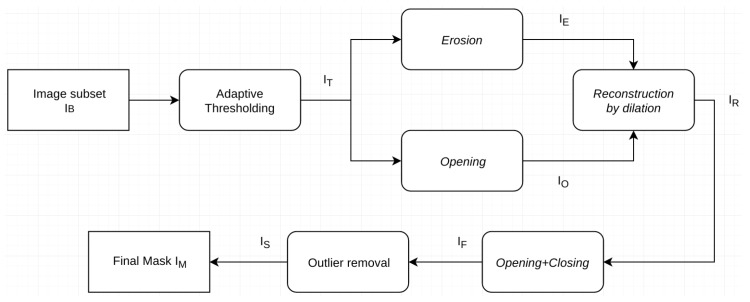
Flowchart of the proposed processing chain.

The novel method for border noise removal that we hereby present is mainly based on mathematical morphology—a well-known theoretical framework for the analysis of spatial structures. The definitions of the *operators* used in this paper can be found in the Appendix A. For all the details on mathematical morphology, the reader can refer to [13]. A flowchart of the proposed processing chain is available in Figure 2.

**Figure 3:**
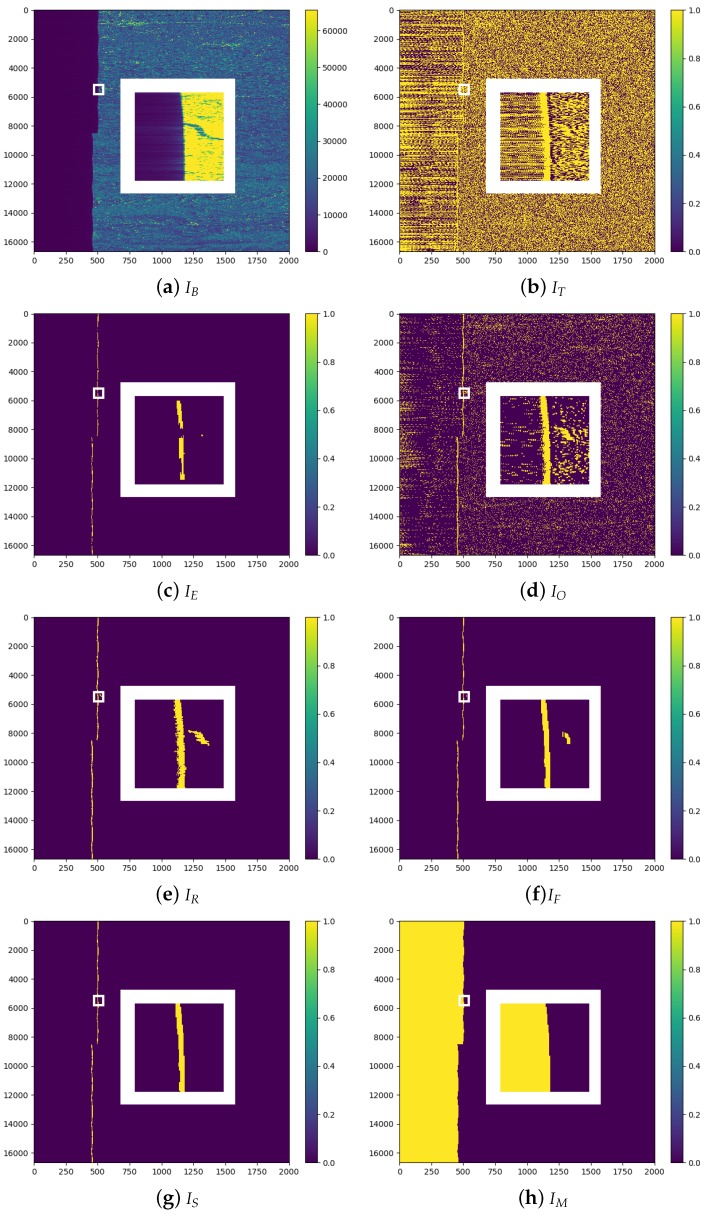
Border noise masking processing steps (overall image and inner white frame showing the close-up of a smaller area around the border noise interface): (**a**) clipping of the input image around the border noise edge; (**b**) adaptive thresholding to detect local discontinuities; (**c**) generation of a *marker* image containing only the border noise/data interface (BDI) pixels; (**d**) generation of a *mask* image to better define the BDI outline; (**e**) *reconstruction by dilation* of the *mask* image using the *marker* pixels as seeds; (**f**) refinement of the BDI outline through a combination of an *opening* and a *closing*; (**g**) removal of outliers; (**h**) final border noise mask in yellow.

For the sake of clarity, the processing steps are hereafter itemized and depicted in Figure 3, showing the overall images and a close-up of a smaller area around the border noise interface to allow better appreciation of the details (white frame).

- First, the input image is clipped to create a subset $I_{B}$ containing the border noise edge, as shown in Figure 3a. This reduces the amount of data to process and the presence of interfaces that are not related to the border noise. According to [4], a safe value for taking into account all the possible noise pixels generated at the border is 2000 samples; therefore, we use this value to define the image width, *W*. In the proposed example, the image has been clipped around the left edge. The same procedure has to be replicated for all of the other possible edges (right, top and bottom).
- Due to the local nature of border noise, the image $I_{B}$ is then binarized through adaptive thresholding [14], which does not compute a global threshold for the whole image, but locally calculates a threshold in regions of small size around each pixel. As the transition from noise to data is relatively sharp, the dimension of the local window is kept small and set to 21 × 21 px (∼0.01 *W*). The obtained binary image $I_{T}$ is depicted in Figure 3b; all the pixels with values below the local threshold have been set to zero (blue), the others to one (yellow). As can be seen, the output is quite noisy (many local discontinuities are found), but the main border noise/data interface (BDI) is visible. Although it is vertically elongated, the BDI outline is not perfectly linear, but it is characterized by a convex shape (mixed with horizontal shifts) that makes difficult to correctly extract it. To address the problem, we designed a set of filters based on mathematical morphology. More specifically, the main *operator* is the *reconstruction by dilation*, which requires us to first define a *marker* image and recursively *dilate* it until the contour of a *mask* image is reached (see Equation (A6)).
- To create the *marker*, we apply an *erosion* (see Equation (A1)) to $I_{T}$. To suitably fit the shape of the BDI, we use, as a *structuring element (SE*), a long vertical line of length $L_{1}$ = 100 px. This means that all the pixel clusters in $I_{T}$ that do not match this shape are discarded. The result of the *erosion* is the binary image $I_{E}$, shown in Figure 3c. As can be seen, almost all of the spurious local interfaces have been removed and the pixels belonging to the BDI are mainly left. However, part of the BDI has also been *eroded* and therefore, it cannot be directly used without defining a *mask*.
- In the *reconstruction* process, the *mask* image is used to delimit the maximum extent that the pixel clusters selected within the *marker* image can reach. In order to better delineate the outline of BDI, this time, we apply to $I_{T}$ an *opening* (see Equation (A3)) by a shorter *SE*, namely, a vertical line of length $L_{2}=0.1L_{1}$, which allows the image to be filtered while retaining more detail. The obtained binary image $I_{O}$ is depicted in Figure 3d.
- Once the *marker* and *mask* images have been generated, it is possible to use $I_{E}$ as a seed to *reconstruct by dilation*$I_{O}$. In this way, a new image $I_{R}$ is created, in which all the pixel clusters shorter (in the vertical direction) than 100 px have been removed and a better defined BDI outline is obtained. The result is depicted in Figure 3e.
- To further refine the BDI outline and remove the undesired horizontal lines and vertical gaps left from the previous steps, two more filters are applied: an *opening* followed by a *closing* (see Equation (A4)). The chosen *SE* is again a vertical line of length 100 px. Figure 3f shows the obtained binary image $I_{F}$.
- In order to cope with the outliers, an additional step is performed that removes the pixel clusters that are too small (area < 1000 px) or too far (more than 3 standard deviations) from the average BDI coordinate along the *x*-axis, generating the binary image $I_{S}$. In the example proposed in Figure 3g, a small cluster of pixels is found and discarded.
- Lastly, all the pixels comprised between the actual image border and the BDI are selected to create the final border noise mask $I_{M}$, shown in yellow in Figure 3h.

## 3. Results

As we discussed earlier, at the moment, no detailed performance assessment of the available border noise removal tools has been ever made. This is mainly due to the difficulty of creating a sufficiently large set of ground truth masks. Thanks to the deployment of IPF version 2.90, this problem has been partially solved. Although the pattern of the border noise in GRD products changes from image to image, we can assume that for two images acquired at the same time on the same orbit within a time interval of 12 days (Sentinel-1 repeat cycle for each satellite), it will be very similar.

**Figure 4:**
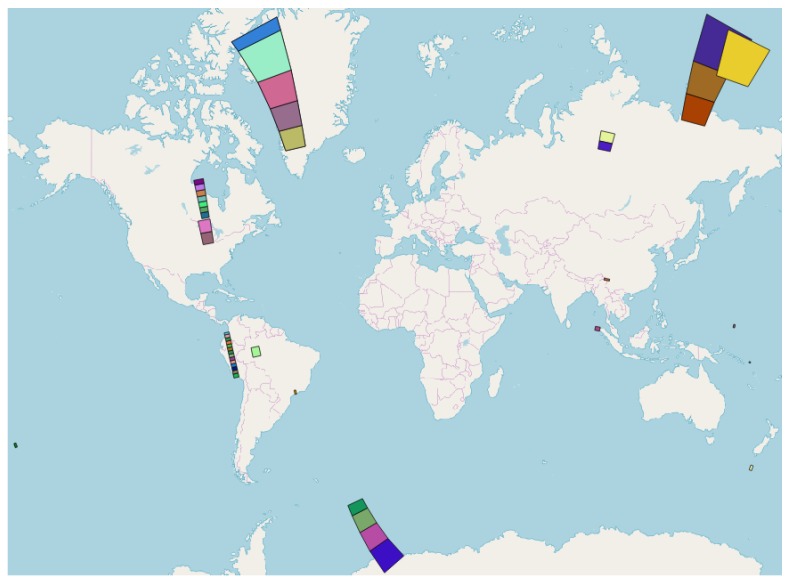
Footprints of the Sentinel-1 products selected for the performance assessment. Base layer ©OpenStreetMap contributors 2018.

To create our dataset, we therefore selected a number of GRD products acquired before the IPF switching date and the corresponding GRD products acquired after that date, namely on 8 March 2018 and 20 March 2018. The former set of images (containing the border noise) was used as the test set, while the latter (noise-free) was used as the ground truth. Nevertheless, for SM products (not concerned by the IPF upgrade), manually created masks were used. In order for the analysis to be as comprehensive as possible, we collected a reasonably large and diversified dataset, containing all the generally available modes, resolutions, and polarizations. Moreover, to deal with a broader range of image scenes and challenging situations, we selected products that were acquired at different latitudes (even comprising the Arctic and Antarctic regions) over both land and water (as well as over land/water transition areas). The footprints of the images are provided in Figure 4, whereas Table 2 reports the exact numbers and types of the products used: 25 IW-H products; 19 EW-M products, 5 EW-H products, and 5 SM-H products. Almost all of the images have two channels (SDH products, containing the HH/HV bands, and SDV products, containing the VV/VH bands), except for four EW single-polarization products (SSH, containing only the HH band). Taking all the bands into account, the total number of images that comprise the dataset is 104.

The assessment consists of a quantitative evaluation of the results provided by the proposed approach (MORP) in comparison with SNAP’s ‘Remove GRD Border Noise’ module (SNAP). The amount of border noise in the original images (ORIG) is also reported in order to evaluate its impact on the quality of the data. To this end, we computed the confusion matrices for ORIG, SNAP, and MORP, considering the classes ‘Noise’ (the pixels to mask, comprising both the border noise pixels and the pixels correctly set to zero) and ‘Data’ (the actual data pixels) and extracted the corresponding Kappa coefficients and commission/omission errors.

**Figure 5:**
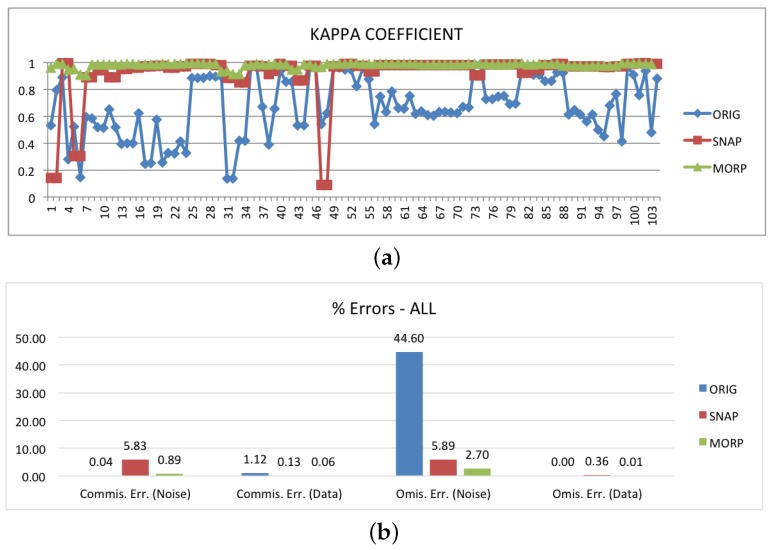
Performance assessment of the proposed method (MORP), compared to the original unmasked images (ORIG) and the images masked via SNAP’s ‘Remove GRD Border Noise’ module (SNAP): (**a**) plot of the Kappa coefficient per single image; (**b**) aggregated commission/omission errors for the entire dataset.

**Figure 6:**
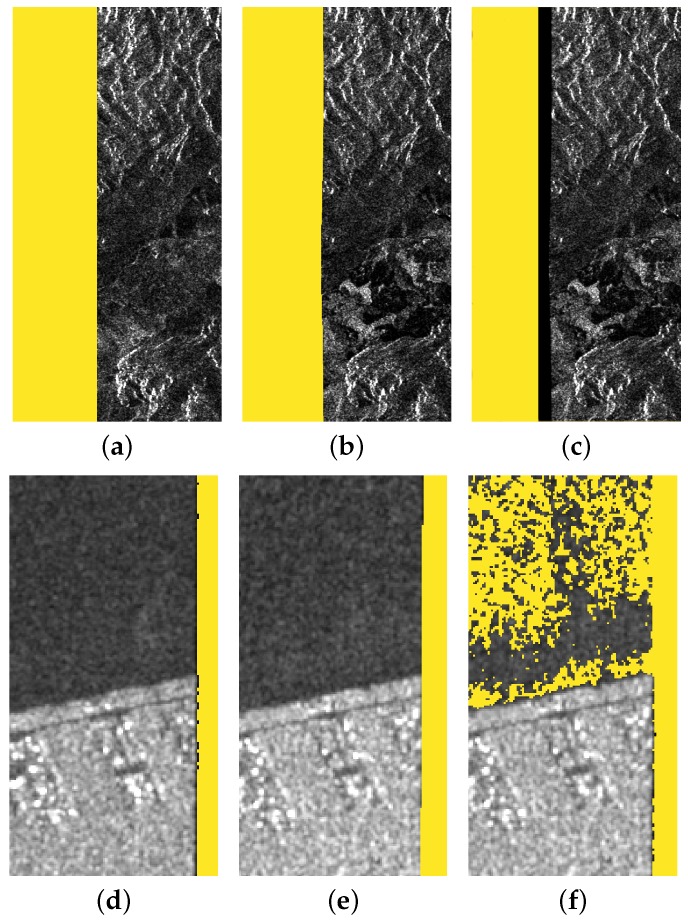
Examples of border noise masks (in yellow) generated by MORP (**b**,**e**) and SNAP (**c**,**f**). In (**a**,**d**), the ground truth masks are provided for comparison. Sentinel-1 images ©Copernicus 2018.

**Figure 7:**
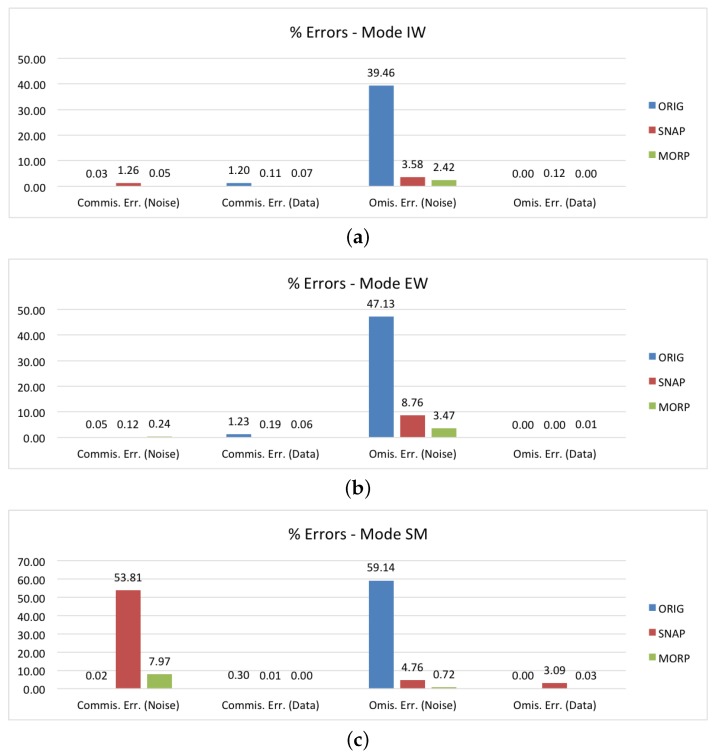
Performance assessment of the proposed method (MORP), compared to the original unmasked images (ORIG) and the images masked via SNAP’s ‘Remove GRD Border Noise’ module (SNAP): (**a**–**c**) commission/omission errors disaggregated by the acquisition mode.

**Figure 8:**
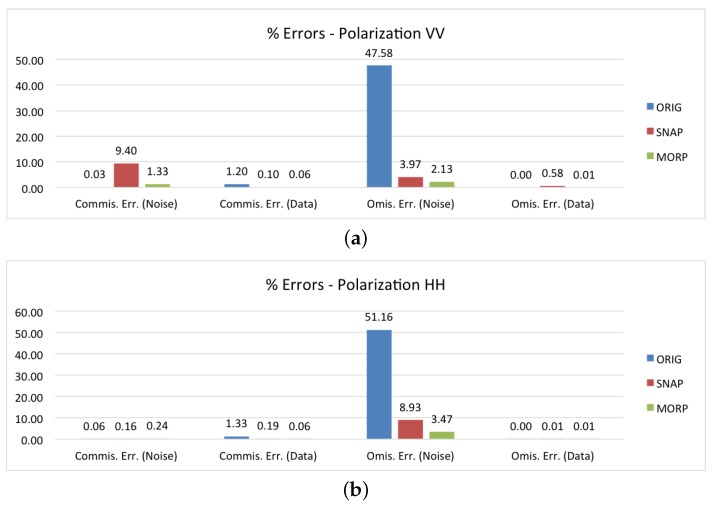

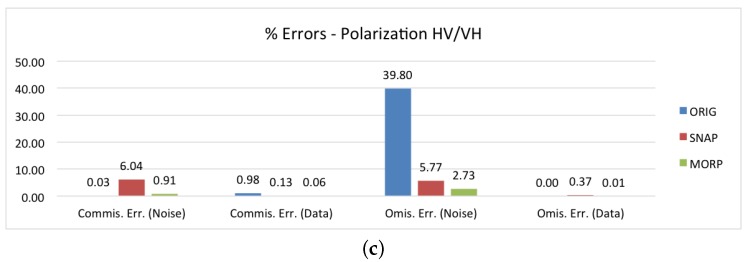
Performance assessment of the proposed method (MORP), compared to the original unmasked images (ORIG) and the images masked via SNAP’s ‘Remove GRD Border Noise’ module (SNAP): (**a**,**c**) commission/omission errors disaggregated by the polarization channel.

**Figure 9:**
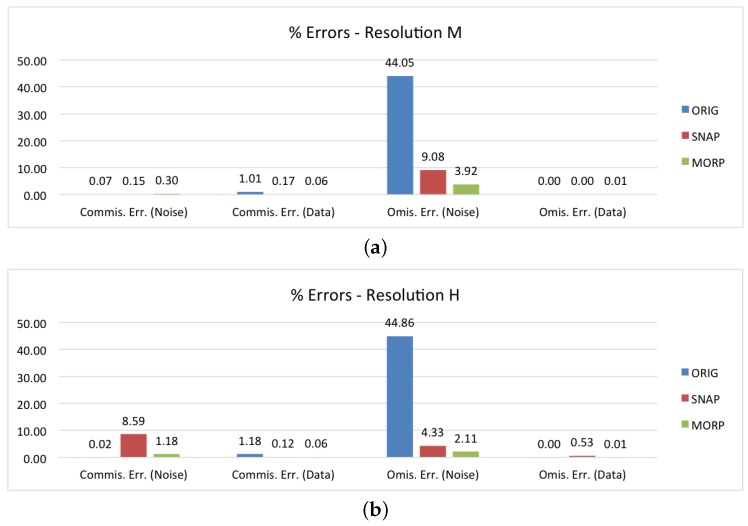
Performance assessment of the proposed method (MORP), compared to the original unmasked images (ORIG) and the images masked via SNAP’s ‘Remove GRD Border Noise’ module (SNAP): (**a**,**b**) commission/omission errors disaggregated by spatial resolution.

The results are shown in Figure 5, Figure 6, Figure 7, Figure 8 and Figure 9. The first graph in Figure 5a reports the Kappa coefficient for each image before any noise border removal (blue curve) and after the application of SNAP (red curve) and MORP (green curve). What can be immediately noticed is that the quality of the original images is very much affected by the amount of border noise. Depending on it, the values of Kappa can range from 0.13 to 0.98, with an average value of 0.67. This shows the importance of correctly and consistently removing the noisy pixels. If we then look into the SNAP and MORP curves, we can clearly see that the latter provides the best performance. In particular, the SNAP curve is characterized by a number of undesired glitches that, in some cases, (six images, corresponding to three products) result in a Kappa coefficient that is even lower than ORIG’s. The whole range of values for SNAP is, in fact, between 0.09 and 0.99, with an average of 0.91, whereas, for MORP, the minimum and maximum Kappa coefficients are 0.90 and 0.99, respectively. On average, MORP allows a Kappa value of 0.98 to be reached.

To understand the reasons behind the low values of Kappa, we have to analyze the ‘Noise’ omission and commission errors reported in Figure 5b. Obviously, the main issue with ORIG is the high omission error—no border noise has been masked, and therefore almost half of the ‘Noise’ class pixels remained unmasked. This number drastically drops off when SNAP and MORP are applied. For SNAP, the average error rate is 5.89%, whereas MORP is capable of reducing it to 2.70%. It should not be a surprise that the omission error is not zero. As we already said, the ground truth images do not perfectly match the images to be masked; therefore, we have to take into account some discrepancies in the results. In particular, not only the number and position of border noise pixels might differ, but also the shape of the BDI can be different. For example, in our dataset, the images processed with IPF 2.90 tend to have straighter interfaces, as can be seen by comparing the border noise masks (in yellow) in Figure 6a,b.

This means that, generally, SNAP and MORP will correctly mask less pixels, but this will result in a higher rate of omission errors. Moreover, in some cases, SNAP is not able to completely remove the border noise, as shown in Figure 6c, where a vertical dark stripe is still present in the masked image. This explains why the omission error is higher than for MORP.

In regard to the commission errors, we have already reported that SNAP is known to fail over water body areas under low wind conditions. In such cases, the water pixels are misclassified as border noise and erroneously masked. This results in a commission error rate of 5.83% for the entire dataset, further reducing the overall accuracy of the method. On the other hand, MORP’s commission error rate remains negligible (0.89%), revealing that this kind of over-masking did not take place.

Along with the overall average errors, to understand if any sort of bias could affect the performance, a confusion matrix was calculated specifically for each mode, polarization, and resolution.

Figure 7a reports the commission and omission errors for the IW images. The percentage of border pixels that are not correctly set to zero in the original images is about 40%. Once MORP and SNAP are applied, this value drops off and is in agreement with the overall average errors. The commission errors are instead lower than those of the overall dataset, especially for SNAP, meaning that the images do not contain any water areas that were misclassfied as border noise.

In Figure 7b, we can find the results for the EW mode. In this case, MORP also reduces the number of unmasked pixels from 47% to 3.5%, and its behaviour seems to be in line with the general results. This does not hold true for SNAP, which certainly removes a large part of the border noise, but keeps almost 9% of pixels unmasked. This is a high value that cannot be explained by the mismatch between the test and ground truth images, but, as we showed before, reveals a failure in the masking algorithm. In regard to the commission errors, they are all negligible.

The results for the third and last mode, SM, are provided in Figure 7c. In terms of omission errors, SNAP is in line with the average results, while for MORP, it is lower than 1%. What should be instead immediately noted is the 53% commission error rate for SNAP. Such a high number clearly indicates a failure of the method. In fact, we can see on the right side of Figure 6f that a large part of the ‘Data’ pixels was masked. As a matter of fact, MORP also experiences a higher commission error rate. In this case, the reason is different, as can be seen by comparing Figure 6d,e that show the ground truth border noise mask and the mask generated via MORP, respectively. As no SM ground truth products are currently available, the mask was manually generated by thresholding the original image. The result is actually not very precise and some border noise pixels along the whole BDI are not removed. This causes the mismatch between the two images and makes the commission error rate increase. In addition, it should be noted that the number of SM images is five times less than the others; thus, the percentage error rate is generally higher, due to the difference in number of pixels analyzed. However, as can be seen in the provided example, the border noise mask generated via MORP is actually very accurate. This result is of particular interest as it provides an effective solution for the processing of SM products, that have been excluded by the IPF upgrade and are still being generated with border noise.

The next three graphs in Figure 8a–c report the errors for the co-polarization (VV and HH) and the cross-polarization (VH/HV) bands. As far as SNAP is concerned, we can observe that the results for the VV channel are mainly biased by the SM products (which are all SDV), producing a commission error rate higher than the average one (9.40%), whereas, for the HH channel, there is a tendency to underestimate the number of border noise pixels (8.93% of omission error). The combination of these two effects is evident in the graph for the cross-polarization, where both high commission and omission errors are present. On the other hand, MORP does not show any specific bias related to polarization, and there does not seem to be any limitations on the selection of the image channel that could be used for the generation of the border noise mask.

The last two graphs provide the performance assessment disaggregated by the image resolution type. In Figure 9a, the results for the medium resolution products are depicted. All these products were acquired in the EW mode; therefore, the percentages are very similar to those in Figure 7b, with a high omission error for SNAP and results in line with the average ones for MORP. In regard to the high resolution products, they are mainly composed of IW and SM images; thus, the graph in Figure 9b shows a high commission error rate (8.59%) for SNAP and no specific issues for MORP.

As the method is intended to be operationally used in Sentinel-1 image pre-processing, the computation time becomes a crucial aspect to take into consideration. We therefore ran MORP (developed in Python) on a machine equipped with an 8-core processor @3.5 GHz and 16 GB memory, and measured the time needed to remove the border noise from all the GRD products in our dataset. The performance was benchmarked against SNAP, run on the same machine via its command-line interface. Table 3 provides the mean, minimum, maximum, and standard deviations of the computation times disaggregated by acquisition mode and resolution (SM-H, EW-M, EW-H, IW-H). In order to allow a direct comparison between single and dual polarization products, the measurements were done separately for each image band (104 in total, see Table 2) and are therefore expressed in seconds per band. As can be seen from column 2, IW-H are the most demanding products, with an average computation time of 51 s, followed by the EW-H and SM-H images, that need 38 s and 34 s, respectively. EW-M images are generally the fastest, requiring 30 s to be processed. This trend can be immediately explained by looking at the last column of the table, which provides the average image sizes and shows a direct relation between computation time and image dimension. If we compare the results with the mean computation times of SNAP (column 3), we can observe that the trends are analogous, although MORP’s averages are roughly twice as high as SNAP’s (the ratios range from 1.66 to 2.87). This difference is certainly due the greater number of operations performed by MORP, but to some extent, can also depend on the software implementation, as the tools have not been written in the same language and use the computer resources differently.

The correlations between the the two methods become less evident if we look at columns 4–9, from which we can derive that, for MORP, the computing times tend to be more dispersed than for SNAP. This means that, along with the image size, the scene features (the number of borders affected by noise, the shape of the border noise interface, etc.) can also contribute to the overall computational complexity, generally increasing it. However, the extra processing time required by MORP with respect to SNAP seems to be acceptable compared to the significant improvements that it would bring in terms of accuracy. As a matter of fact, introducing into the full Sentinel-1 pre-processing chain (thermal noise removal, calibration, terrain correction, etc.) an additional block that, on average, would increase the processing time by less than two minutes (for the larger products) should not significantly degrade the final computational efficiency.

## 4. Conclusions

In this paper, we have proposed a novel method based on mathematical morphology for the automatic detection and masking of border noise in Sentinel-1 GRD products. The goal is to overcome the limitations of the currently available techniques for border noise removal and provide an operational tool that is able to process any GRD image regardless of its acquisition mode, polarization, or resolution. To this end, we carried out a detailed performance assessment that proved the effectiveness of the proposed method in drastically improving the quality of the original images. A direct comparison with the results provided by SNAP’s ‘Remove GRD Border Noise’ module also showed that the method does not seem to be affected by any specific bias related to the Sentinel-1 acquisition parameters or to challenging features in the scene that might be misdetected as border noise. In terms of computational efficiency, although, on average, it was shown to be two times slower than SNAP, the proposed method is capable of processing the largest products (IW, high resolution) in less than two minutes, therefore being suitable for operational use.

## Figures and Tables

**Table 1:** Summary of the characteristics of the main Ground Range Detected (GRD) products.

Mode	Resolution [rg × az]		Pixel Spacing [rg × az]		No of Looks		ENL		Swath Width
	**M**	**H**	**M**	**H**	**M**	**H**	**M**	**H**	
**SM**	84 × 84 m	23 × 23 m	40 × 40 m	10 × 10 m	22 × 22 m	6 × 6 m	398.4 m	29.7 m	80 Km
**EW**	93 × 87 m	50 × 50 m	40 × 40 m	25 × 25 m	6 × 2 m	3 × 1 m	10.7 m	2.7 m	410 Km
**IW**	88 × 87 m	20 × 22 m	40 × 40 m	10 × 10 m	22 × 5 m	5 × 1 m	81.8 m	4.4 m	250 Km

**Table 2:** Dataset details.

Mode		SM			EW			IW	
Resolution		M	H		M	H		M	H
	**SSH**	-	-		4	-		-	-
**Polarization**	**SDH**	-	-		13	5		-	-
	**SDV**	-	5		2	-		-	25
**No. products**		5			24			25	
**No. images**		10			44			50	

**Table 3:** Performance assessment of the proposed method (MORP) in comparison with SNAP’s ‘Remove GRD Border Noise’ module (SNAP): mean, minimum, maximum, and standard deviation of the computation time required to process a single image band. The results are disaggregated by mode and resolution.

Mode		Computation Time [s/band]											Avg Image Size [px]
		**Mean**			**Min**			**Max**			**Std Dev**		
		**MORP**	**SNAP**		**MORP**	**SNAP**		**MORP**	**SNAP**		**MORP**	**SNAP**	
**SM-H**		33.66	11.72		12.92	9.16		44.77	13.54		12.10	1.61	10,805${}^{2}$
**EW-M**		29.72	11.24		19.80	7.51		52.66	13.61		8.86	1.63	10,112${}^{2}$
**EW-H**		37.78	17.88		36.91	9.73		40.01	21.13		1.32	4.68	14,875${}^{2}$
**IW-H**		50.60	30.35		40.05	16.64		102.43	39.05		18.23	5.38	20,529${}^{2}$

## Appendix A. Mathematical Morphology

Mathematical morphology is a theoretical framework that is used for the analysis of the shapes and forms of objects based on set theory, lattice algebra, and topology [13]. Hereafter, the definitions of the morphological operators used in this paper are provided.

- *Erosion.* The erosion of an image *I* by a structuring element *E* is defined as the locus of points p such that *E* is included in *I* when its origin is placed at p:
  (A1) $$\varepsilon_{E}\left(I\right)=\left\{p\;|\;E_{p}\;\subseteq \;I\right\}.$$
- *Dilation.* The dilation of an image *I* by a structuring element *E* is defined as the locus of points p such that *E* hits *I* when its origin coincides with p:
  (A2) $$\delta_{E}\left(I\right)=\left\{p\;|\;E_{p}\;\cap \;I\neq ⊘\right\}.$$
- *Opening.* The opening of an image *I* by a structuring element *E* is defined as the erosion of *I* by *E* followed by the dilation with the reflected $\overset{˘}{E}$:
  (A3) $$\gamma_{E}\left(I\right)=\delta_{\overset{˘}{E}}\left[\varepsilon_{E}\left(I\right)\right].$$
- *Closing.* The closing of an image *I* by a structuring element *E* is defined as the dilation of *I* by *E* followed by the erosion with the reflected $\overset{˘}{E}$:
  (A4) $$\phi_{E}\left(I\right)=\varepsilon_{\overset{˘}{E}}\left[\delta_{E}\left(I\right)\right].$$
- *Geodesic Dilation.* The geodesic dilation of size 1 of a marker image $I_{mk}$ with respect to a mask image $I_{ms}$ (with $I_{mk}\leq I_{ms}$) is defined as the point-wise minimum between the mask and the dilation by the elementary isotropic structuring element of the marker:
  (A5) $$\delta_{ms}^{\left(1\right)}\left(I_{mk}\right)=\delta \left(I_{mk}\right)\wedge I_{ms}.$$
- *Reconstruction by Dilation.* The reconstruction by dilation of a mask image $I_{ms}$ from a marker image $I_{mk}$ (with $I_{mk}\leq I_{ms}$) is defined as the geodesic dilation of $I_{mk}$ with respect to $I_{ms}$ iterated until stability:
  (A6) $$\begin{array}{cc} R_{I_{ms}}^{\delta }\left(I_{mk}\right)=\delta_{I_{ms}}^{\left(i\right)}\left(I_{mk}\right), & \text{where}\;i\;\text{is such that}\;\delta_{I_{ms}}^{\left(i\right)}\left(I_{mk}\right)=\delta_{I_{ms}}^{\left(i+1\right)}\left(I_{mk}\right) \end{array}.$$
